# Effects of mRNA expression of five Notch ligands on prognosis of gastric carcinoma

**DOI:** 10.1038/s41598-022-19291-5

**Published:** 2022-09-07

**Authors:** Yunlong Li, Fengni Xie, Huimin Zhang, Xiao Wu, Gang Ji, Jipeng Li, Liu Hong

**Affiliations:** Present Address: Department of Gastrointestinal Surgery, The First Affiliated Hospital of Air Force Medical University, Xi’an, 710032 Shaanxi Province China

**Keywords:** Cancer, Gastrointestinal cancer, Oncogenes, Tumour biomarkers

## Abstract

Notch ligands are expression changes in a great many malignancies including gastric cancer (GC) frequently. The prognostic value of each Notch ligands in GC patients remains lack of large sample data results. In present research, we researched the prognostic value of Notch ligands in GC patients in order to fill the shortage areas. We used an online database (http://kmplot.com/analysis/index.php?p=service&cancer=gastric) to identify the relationship between mRNA expression of each Notch ligand and overall survival (OS) in GC. We analyze the relevance of overall survival and clinical data which includes gender, Lauren's classification, differentiation, clinical stage and treatment. The study found that high DLL1, DLL3, DLL4 and JAG2 mRNA expression were tied to worse OS in all GC patients followed up for 10 years. There is no significant relevance to the expression of JAG1 mRNA and OS in patients with GC. We also did a survey of each Notch ligands in different clinical and pathological features present different prognosis. The information will help to better understand the biology of gastric cancer heterogeneity, provide more accurate prognostic evaluation tools and provide new targets for targeted drug development besides.

## Introduction

Worldwide, the latest statistics show that the cancer-related death for gastric cancer (GC) was the third-highest percentage (around 8.2% of cancer-related deaths each year globally)^1^. Early detection is still a worldwide problem and the diagnosis of GC generally is locally advanced. Patients with advanced GC still present poor prognosis despite the advances in radical cure operation such as Laparoscopic surgery or Da Vinci Robotic surgery and multimodal therapeutic modalities after surgery^2,3^. Therefore, to clarify the molecular mechanism of the occurrence and development of GC, finding prognostic biomarkers and targets will help provide individualized treatment, to cut down the chance of postoperative recurrence and enhance clinical outcome. According to research findings, the Notch signaling pathway was one of the crucial factors therapeutic targets for cancers. The Notch affects patient outcome by regulating cell proliferation, apoptosis, invasion and angiogenesis^4,5^. The Notch includes four receptors (Notch 1-4) and five ligands (DLL1, -3, -4 and JAG1, -2). New research has found that once the Notch signal is activated, it plays a pivotal role in the occurrence and development of GC as well as interact with other intercellular signaling pathways, participating in inhibition of apoptosis, metastasis and chemotherapy resistance^6,7^. So far, the effect of Notch receptor mRNA level on OS of patients with non-small cell lung cancer and GC has been reported^8–10^.

Nevertheless, the effect of individual Notch ligand mRNA level on prognosis in patients with GC hasn't been reported. We used "Kaplan–Meier Plotter" (KM Plotter) to analyze the expression of Notch ligand in GC patients and its effect on prognosis. The KM plotter can analyze survival of 876 GC patients combined with clinical data and gene expression (http://kmplot.com/analysis/index.php?p=service&cancer=gastric), dealt with by a PostgreSQL server. Up to now, four receptors of Notch have been reviewed and analyzed by KM plotter in GC^11,12^. The objective of the study was: to analyze the prognostic effects of mRNA expression of 5 Notch ligand in 876 patients with GC using the KM plotter database.

## Materials and methods

We used an online database to identify the correlation factor of individual Notch ligands expression to the OS in GC, analyze the relevance of OS and clinical data, which is included in gender, clinical stage, differentiation, Lauren's classification and treatment. All cancer patients in the database were identified from The Cancer Genome Atlas (TCGA, http://cancergenome.nih.gov), the Gene Expression Omnibus (GEO, http://www.ncbi.nlm.nih.gov/geo/) and The European Genome-phenome Archive (EGA, https://ega-archive.org/datasets). A database was set up using ten-year follow-up data and gene expression data of 876 carcinoma of stomach downloaded from GEO. The specific datasets are GSE14210, GSE15459, GSE22377, GSE29272, GSE51105 and GSE62254. In another word, five Notch ligands were entered into the database ((http://kmplot.com/analysis/index.php?p=service&cancer=gastric) to acquire Kaplan–Meier survival plots. In Kaplan–Meier Plotter, three Probe set options are provided. This study we chose only JetSet best probe set. Jetset selects the optimal probe set for each gene using a scoring method established to assess each probe set for specificity, coverage and degradation resistance. When auto select best cutoff is selected, all possible cutoff values between the lower and upper quartiles are computed, and the best performing threshold is used as a cutoff. In general, p value < 0.05 were considered statistically significant.

### Statement

This research was performed in accordance with relevant guidelines/regulations, and informed consent was obtained from all participants and/or their legal guardians. This Research have been performed in accordance with the Declaration of Helsinki.

## Results

**Figure 1:**
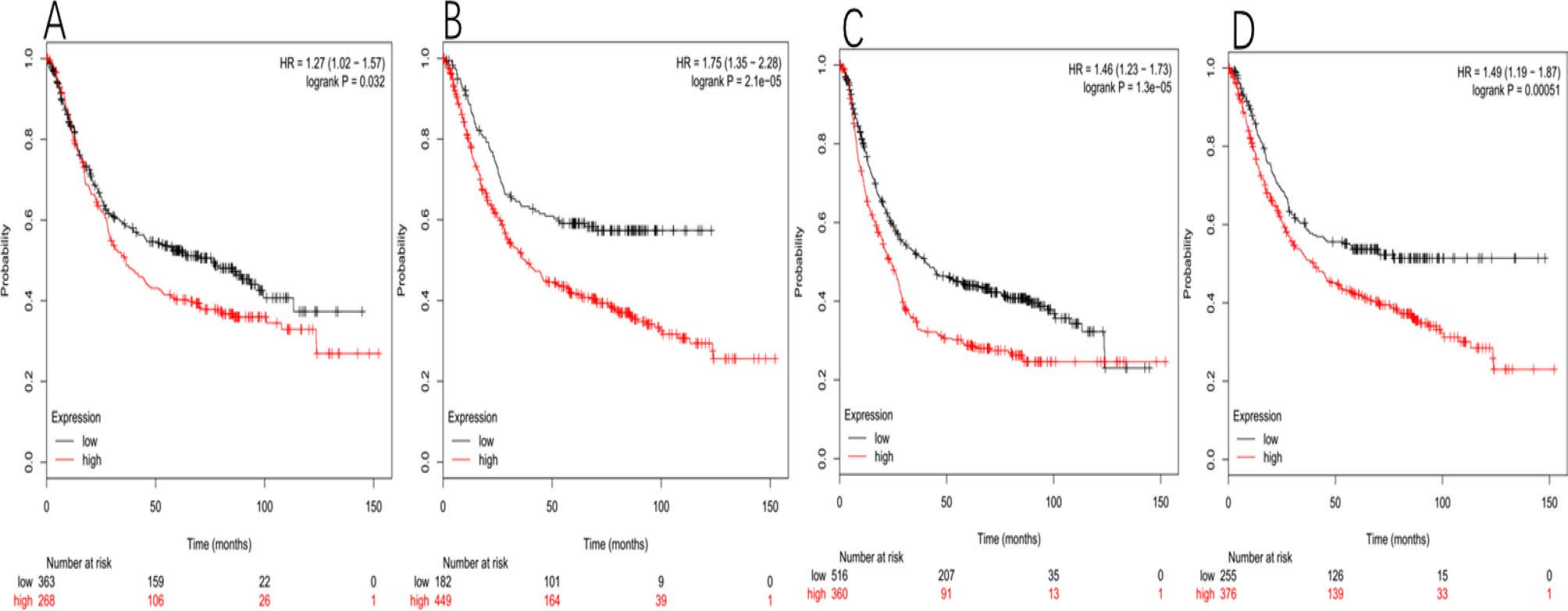
For DLL1, The Affymetrix ID is: 224215_s_at. (**A**) OS curves are plotted for GC patients (n = 631). The Affymetrix ID is: 227938_s_at. (**B**) OS curves are plotted for GC patients (n = 631). For DLL3, The Affymetrix ID is: 219537_x_at. (**C**) OS curves are plotted for GC patients (n = 876). The Affymetrix ID is: 222898_s_at. (**D**) OS curves are plotted for GC patients (n = 631).

Kaplan–meier survival data of GC patients with differential expressions of Notch ligand can be discovered at www.kmplot.com. We were the first to analyze the prognostic value of DLL1 expression. The Affymetrix ID is: 224215_s_at. Figure 1A shows the survival curve of GC patients based on DLL1 mRNA expression level (n = 631). The ten-year survival rate of all GC patients with high expression level of DLL1 mRNA was worse, HR 1.27 (1.02–1.57), p = 0.032. The Affymetrix ID is: 227938_s_at. OS curves were plotted for carcinoma of stomach patients (n = 631) (Fig. 1B). The ten-year survival rate of all GC with high expression level of DLL1 was also worse, HR 1.75 (1.35–2.28), p = 2.1e−05. For DLL3, the Affymetrix ID is: 219537_x_at. The OS of GC patients with high expression level of DLL3 mRNA was worse, HR 1.46 (1.23–1.73), p = 1.3e−05 (Fig. 1C), the same as the Affymetrix ID is: 222898_s_at, HR 1.49 (1.19–1.87), p = 0.00051 (Fig. 1D).

**Figure 2:**
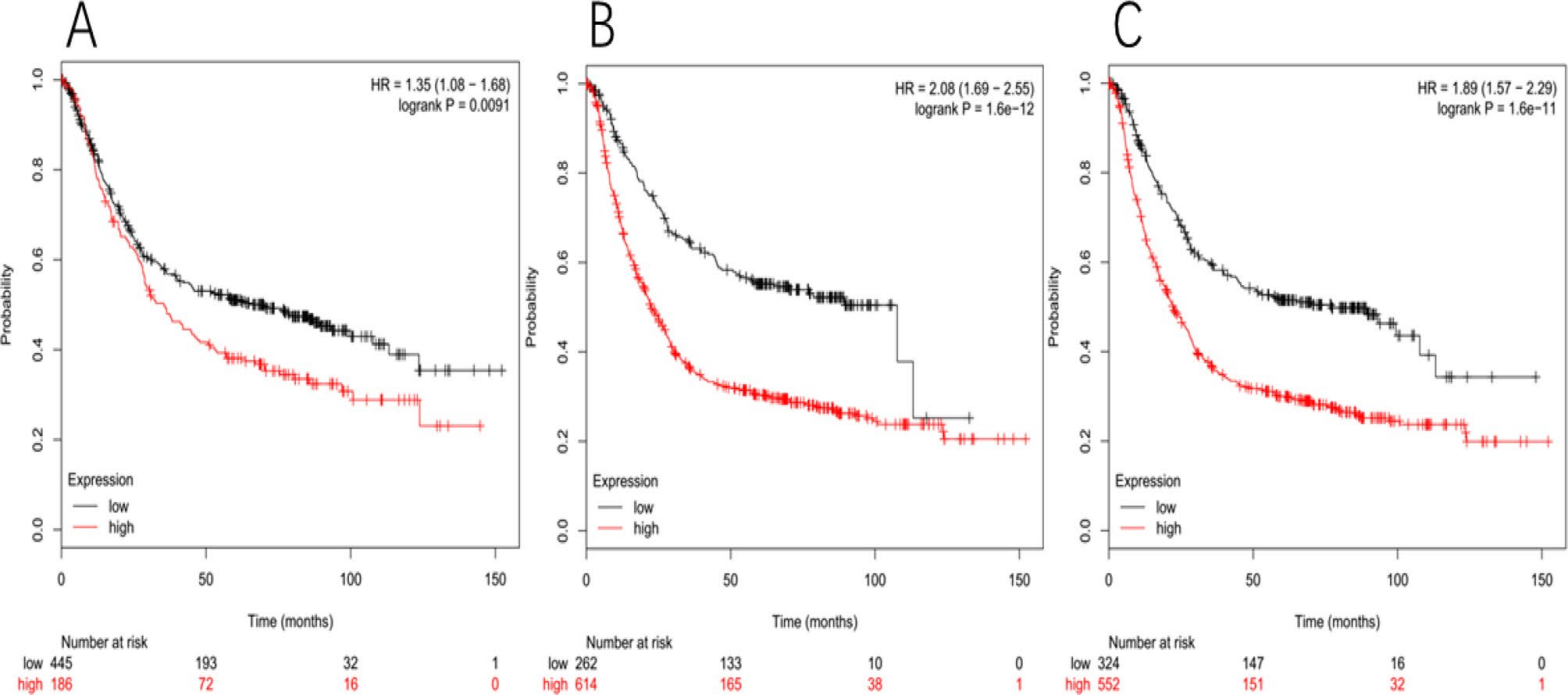
For DLL4, The Affymetrix ID is: 223525_at. (**A**) OS curves are plotted for GC patients (n = 631). For JAG2, The Affymetrix ID is: 209784_s_at. (**B**) OS curves are plotted for GC patients (n = 876). The Affymetrix ID is: 32137_at. (**C**) OS curves are plotted for GC patients (n = 876).

For DLL4, the Affymetrix ID is: 223525_at. High expression of DLL4 was significantly tied to worsening OS in GC patients, HR 1.35 (1.08–1.68), p = 0.0091 (Fig. 2A). For JAG2, the Affymetrix ID is: 209784_s_at. The OS of GC patients with high expression level of JAG2 mRNA was worse, HR 2.08 (1.69–2.55), p = 1.6e−12 (Fig. 2B). The same as Affymetrix ID is: 32137_at. HR 1.89 (1.57–2.29), p = 1.6e−11 (Fig. 2C).

**Figure 3:**
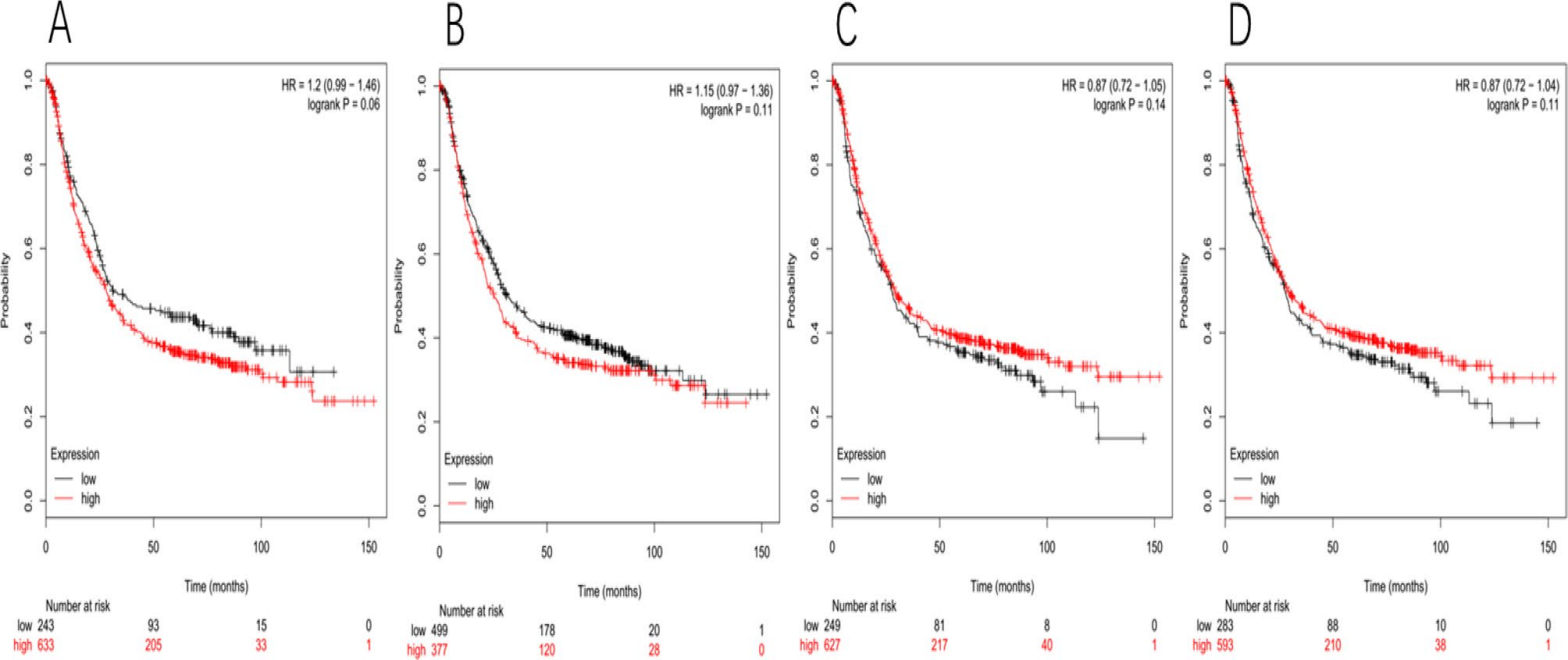
For JAG1, The Affymetrix ID is: 216268_s_at. (**A**) OS curves are plotted for GC patients (n = 876). The Affymetrix ID is: 209098_s_at. (**B**) OS curves are plotted for GC patients (n = 876). The Affymetrix ID is: 209099_x_at. (**C**) OS curves are plotted for GC patients (n = 876). The Affymetrix ID is: 216268_s_at. (**D**) OS curves are plotted for GC patients (n = 876).

For JAG1, the Affymetrix ID is: 216268_s_at. High expression of JAG1 was insignificant correlation with worsening OS in GC patients, HR 1.2 (0.99–1.46), p = 0.06 (Fig. 3A). Also, the same as the Affymetrix ID: 209098_s_at (Fig. 3B), the Affymetrix ID is: 209099_x_at (Fig. 3C) and the Affymetrix ID is: 216268_s_at (Fig. 3D).

We filled the gaps in the association between individual Notch ligands and clinicopathological features (Table 1). Just as, DLL3 high expression was so clearly tied to worsening OS in male, HR 1.71 (1.38–2.14), p = 1.2e−6. DLL4 mRNA expression was so clearly tied to worsening OS in male, HR 1.43 (1.06–1.95), p = 0.02. JAG2 mRNA expression was so clearly tied to worsening OS in male, HR 2.12 (1.70–2.64), p = 7.3e−12 and female as well, HR 1.77 (1.24–2.52), p = 0.002. High expression of DLL3 was tied to worsen OS in advanced GC, such as stage II, HR 2.37 (1.23–4.55), p = 0.008 and stage III, HR 1.56 (1.12–2.19), p = 0.009. Nevertheless, DLL3 expression was linked with a better OS in grade I, HR 0.29 (0.11–0.79), p = 0.01. DLL4 expression was only tied to worsening OS in stage IV, HR 1.49 (1.01–2.22), p = 0.045. JAG1 mRNA expression was so clearly tied to a better OS in stage I, HR 0.33 (0.12–0.91), p = 0.025. JAG2 expression was so clearly tied to worsening OS in stage I, HR 3.58 (1.32–9.69), p = 0.007, stage III, HR 2.09(1.53–2.84), p = 1.8e−6 and stage IV, HR 1.68 (1.14–2.47), p = 0.009. DLL1 mRNA expressions was tied to worsening OS in diffuse, HR 0.67 (0.45–1.00), p = 0.048. DLL3 mRNA expressions was tied to worsening OS in intestinal, HR 1.6 (1.02–2.57), p = 0.048. HR 1.88 (1.34–2.64), p = 0.0002. DLL4 expression was so clearly tied to worsening OS in diffuse, HR 1.5 (1.06–2.13), p = 0.021. High expression of JAG1 was linked to improving OS in intestinal, HR 0.63 (0.46–0.86), p = 0.004 and mixed, HR 0.16 (0.03–0.73), p = 0.008. JAG2 mRNA expression was so clearly tied to worsening OS in intestinal, HR 2.72 (1.97–3.74), p = 1.8e-10 as well as diffuse, HR 1.66 (1.16–2.38), p = 0.005. DLL3 expression was tied to a better OS in low differentiation adenocarcinoma, HR 0.61 (0.39–0.94), p = 0.024. However, with worsen OS in high differentiation adenocarcinoma, HR 2.57 (1.03–6.39), p = 0.036. JAG1 expression was tied to a better OS in low differentiation adenocarcinoma, HR 0.59 (0.38–0.91), p = 0.017 and high differentiation adenocarcinoma, HR 0.18 (0.04–0.79), p = 0.011. JAG2 mRNA expression was tied to worsening OS in high differentiation adenocarcinoma, HR 3.66 (1.32–10.15), p = 0.008. DLL3 mRNA expression was tied to worsening OS in surgery combined with 5-FU chemotherapy, HR 1.86 (1.30–2.67), p = 0.0006. DLL4 and JAG2 mRNA expression was so clearly tied to worsening OS in Surgery, same as surgery combined with 5-FU chemotherapy, p < 0.05. JAG1 expression was just tied to worsening OS in Surgery, HR 1.35 (1.01–1.8), p = 0.042.

**Table 1:** Correlation of Notch ligands with OS of GC patients by different clinicopathological parameters.

Clinicopathological parameters	DLL1		DLL3		DLL4		JAG1		JAG2	
	HR (95% CI)	p	HR (95% CI)	p	HR (95% CI)	p	HR (95% CI)	p	HR (95% CI)	p
**Gender**										
Male	1.26 (0.94–1.70)	0.123	1.71 (1.38–2.14)	1.2e−6	1.43 (1.06–1.95)	0.020	0.86 (0.69–1.07)	0.167	2.12 (1.70–2.64)	7.3e−12
Female	0.64 (0.38–1.07)	0.083	0.81 (0.55–1.20)	0.298	1.29 (0.84–2.00)	0.245	0.75 (0.53–1.06)	0.103	1.77 (1.24–2.52)	0.002
**Clinical stages**										
1	3.27 (0.87–11.7)	0.073	0.29 (0.11–0.79)	0.010	0.65 (0.22–1.94)	0.434	0.33 (0.12–0.91)	0.025	3.58 (1.32–9.69)	0.007
2	0.51 (0.22–1.16)	0.101	2.37 (1.23–4.55)	0.008	1.45 (0.77–2.72)	0.243	0.59 (0.32–1.07)	0.081	1.78 (0.95–3.31)	0.066
3	0.70 (0.48–1.02)	0.062	1.56 (1.12–2.19)	0.009	1.4 (0.91–2.17)	0.128	0.75 (0.56–0.10)	0.052	2.09 (1.53–2.84)	1.8e−6
4	0.82 (0.55–1.23)	0.338	0.75 (0.51–1.12)	0.157	1.49 (1.01–2.22)	0.045	1.19 (0.81–1.75)	0.370	1.68 (1.14–2.47)	0.009
**Lauren classification**										
Intestinal	1.6 (1.02–2.57)	0.068	1.88 (1.34–2.64)	0.0002	0.81 (0.56–1.16)	0.253	0.63(0.46–0.86)	0.004	2.72 (1.97–3.74)	1.8e−10
Diffuse	0.67 (0.45–1.00)	0.048	0.72 (0.48–1.09)	0.125	1.5 (1.06–2.13)	0.021	1.38 (0.95–2.02)	0.089	1.66 (1.16–2.38)	0.005
Mixed	3.2 1(0.71–14.54)	0.110	0.43 (0.14–1.27)	0.114	4.18 (0.91–19.29)	0.047	0.16 (0.03–0.73)	0.008	2.54 (0.89–7.26)	0.071
**Differentiation**										
Low differentiation adenocarcinoma	0.67 (0.38–1.17)	0.158	0.61 (0.39–0.94)	0.024	1.31 (0.78–2.19)	0.310	0.59 (0.38–0.91)	0.017	1.57 (0.95–2.6)	0.073
Median differentiation adenocarcinoma	1.47 (0.71–3.04)	0.299	1.93 (0.98–3.77)	0.052	0.78 (0.41–1.5)	0.463	0.62 (0.28–1.35)	0.224	2.06 (0.98–4.32)	0.051
High differentiation adenocarcinoma	None	0.221	2.57 (1.03–6.39)	0.036	None	0.221	0.18 (0.04–0.79)	0.011	3.66 (1.32–10.15)	0.008
**Treatment**										
Surgery	1.30 (0.91–1.85)	0.147	1.25 (0.93–1.66)	0.135	1.47 (1.06–2.04)	0.019	1.35 (1.01–1.8)	0.042	1.54 (1.15–2.05)	0.003
Surgery combined with 5-FU chemotherapy	0.63 (0.23–1.69)	0.354	1.86 (1.30–2.67)	0.0006	2.92 (1.17–7.28)	0.016	0.77 (0.54–1.08)	0.128	1.92 (1.34–2.76)	0.0003

## Discussion

The four receptors and five ligands constituted the Notch signaling pathway, a kind of intercellular communication system which is vitally important for embryogenesis and tissue homeostasis^13^. Current research has shown that DLL1 obviously has a crucial impact on tumorigenesis and the development of many cancers. The most widely studied and comparatively mature is breast cancer, DLL1 overexpression leads to poor prognosis through cell proliferation, maintenance of tumor stem cells and angiogenesis^14–16^. While, clustered DLL1 therapy results in significant enhancement of tumor antigen-specific T cell immune responses and memory, remarkably increased tumor infiltration by T cells, attenuated tumor vascularization, and suppression of tumor growth and produced remarkably enhanced progression-free survival^17^. These articles have been confirmed that DLL1 plays a crucial role in the formation of blood vessels. During the formation of new blood vessels, arterial endothelial cells induce a large amount of DLL1 expression. Angiogenesis in GC is formed under pathological conditions, and it is still regulated by molecular signals similar to the formation of blood vessels under physiological conditions. In this report, by analyzing 10-year follow-up data of GC, we were pleasantly surprised that patients with high expression of DLL1 had a worsening prognosis. Therefore, it also indirectly proved that DLL1 has great potential in tumor therapy of the formation of blood vessels. High expression of DLL1 leads to poor prognosis in patients with diffuse GC. This is consistent with the research conclusion of Giulia's^18^, Notch1 activity in gastric cancer is controlled by the epigenetic silencing of the ligand DLL1, and that Notch1 inhibition is associated with the diffuse type of gastric cancer.

DLL3 is part of the Notch five transmembrane ligands family and presumably a crucial predictive biomarker and therapeutic target^19^. It has been reported that it has a pivotal role in gastrointestinal neuroendocrine carcinoma cells. There is a significant upregulation of DLL3 in gastrointestinal neuroendocrine carcinoma cell lines. The gene silencing of DLL3 caused significant growth inhibition through the induction of intrinsic apoptosis^20^. High-grade neuroendocrine carcinomas were negative for DLL3, and five-year OS and PFS were significantly improved in patients who received adjuvant chemotherapy. However, the patients with positive for DLL3 were no difference^21^. In vitro experiment have been confirmed DLL3 overexpression can promote the proliferation of gastric cancer cells in vitro, and down-regulation of DLL3 inhibits the proliferation of gastric cancer cells^22^. However, the literature we found only a few data have analyzed the relationship between DLL3 and prognosis in gastrointestinal tumors, and the expression of DLL3 in GC and its effect on prognosis have not been reported. In the study, we detect the expression of DLL3 was linked to worsening OS for GC patients followed for ten years, as well as in male patients, in intestinal, surgery, combined with 5-FU chemotherapy and stage II and stage III. These results could herald DLL3 as a negative regulator and an independent biomarker of negative prognosis in advanced GC.

The expression of DLL4 ligand in the Notch signaling pathway is most important in tumor endothelial cells. In the tumor microenvironment, tumor secreted angiogenic factors such as VEGF can induce the expression of DLL4 in endothelial cells^23^. According to the analysis of 383 patients with GC, the overexpression of DLL4 activates the Notch-1 signaling pathway, effectively promotes proliferation, lymph node metastasis and distant metastasis, remarkably increasing resistance to 5-FU chemotherapy^24^. This study verifies that DLL4 expression was meaningfully tied to worsening OS in GC patients for 10 years, as well as male patients, stage and diffuse cancer patients. Unfortunately, overexpression of DLL4 was linked to OS worsening in 5-FU based adjuvant. These results are fully in line with the conclusion of Miao et al. In addition, new research suggests that about 19.9% of GC patients have high expressions of DLL4, and the probability of recurrence and metastasis after radical surgery is high^25^. In summary, it can be said that DLL4 is a potential novel prognostic indicator and therapeutic target for GC to guide clinical practice.

There is now compelling evidence that overexpression of JAG1 can promote metastasis of colorectal carcinoma by inducing epithelial-mesenchymal transition. JAG1 can be used as a target for anti-tumor therapy^26,27^. In contrast, JAG1 has been deemed a tumor suppressor gene in acute myeloid leukemia, which cancer cell growth can be reduced and patients with JAG1 overexpression also have a better prognosis^28^. Xiao’s reported that when miR-124 overexpression in GC the expression of the JAG1 and EZH2 was downregulated, and silencing of JAG1 or EZH2 by RNA interference also suppressed GC cell growth and metastasis. It has been certified that the JAG1 mRNA overexpressions were found in low differentiation adenocarcinoma, samples with lymph node metastasis, and samples at stage II, II and IV in GC tissue^29^. In our data, we found that high JAG1 expression was associated with worsening OS for 10 years of follow-up in GC patients. JAG1 overexpression was tied to better OS in stage I, intestinal, poorly differentiated and well differentiated cancer presents. However, patients treated alone with surgery had a worse prognosis. Our findings complement Xiao's results, but there are some notable differences. The latest research reported one of Notch ligand, JAG2 correlated to several carcinogenesis. In GC, Kang’s reported that JAG2 was significantly higher in GC tissues than in pericarcinomatous tissue. High relative expression of JAG2 was tied to better OS in univariate analysis^30^. Our study shows that for ten years, JAG2 mRNA expression was found to be significantly related to worsening OS for GC patients. JAG2 mRNA overexpression was tied to worsen OS in all clinical stages except stage II. It was inconsistent with Kang's findings. The reason may be due to the difference in sample size. Although this study is the largest sample size reported to date. With the continuous updating and improvement of the database, we believe that there will be a larger sample size to verify our views in the future. Most studies currently focus on the biological effects of a single notch ligand. Whether the expression of the five Notch ligands is related to each other and affects each other is still unknown, especially in tumor cells, which needs to be explored in the future.

In conclusion, by exploiting the KM plotter database, we clear and definite the prognostic roles of five Notch ligands in GC, and update gene expression data and survival information from the largest sample library to date of 876 GC patients. In all patients with GC, there is an insignificant correlation between the expression of only JAG1 mRNA and OS. Our study preliminarily explored the prognostic role of Notch ligand in various clinicopathological characteristics. The results of the research will be useful for understanding the biology of GC heterogeneity, providing more accurate prognostic evaluation tools, and providing new potential targets for targeted drug development.

## Data Availability

The datasets generated during and/or analysed during the current study are available in the [Kaplan–Meier plotter] repository, [(http://kmplot.com/analysis/index.php?p=service&cancer=gastric].
